# Prognostic impact of MRI-based cervical skeletal muscle mass on survival in parotid gland carcinoma

**DOI:** 10.1093/jjco/hyag033

**Published:** 2026-03-02

**Authors:** Ken Kasahara, Takuya Mikoshiba, Katsuyoshi Idei, Yuki Matsui, Takeyuki Kono, Mariko Sekimizu, Hiroyuki Ozawa

**Affiliations:** Department of Otolaryngology and Head and Neck Surgery, Keio University School of Medicine, 35 Shinanomachi, Shinjuku-ku, Tokyo 160-8582, Japan; Department of Otolaryngology and Head and Neck Surgery, Keio University School of Medicine, 35 Shinanomachi, Shinjuku-ku, Tokyo 160-8582, Japan; Department of Otolaryngology and Head and Neck Surgery, Keio University School of Medicine, 35 Shinanomachi, Shinjuku-ku, Tokyo 160-8582, Japan; Department of Otolaryngology and Head and Neck Surgery, Keio University School of Medicine, 35 Shinanomachi, Shinjuku-ku, Tokyo 160-8582, Japan; Department of Otolaryngology and Head and Neck Surgery, Keio University School of Medicine, 35 Shinanomachi, Shinjuku-ku, Tokyo 160-8582, Japan; Department of Otolaryngology and Head and Neck Surgery, Keio University School of Medicine, 35 Shinanomachi, Shinjuku-ku, Tokyo 160-8582, Japan; Department of Otolaryngology and Head and Neck Surgery, Keio University School of Medicine, 35 Shinanomachi, Shinjuku-ku, Tokyo 160-8582, Japan

**Keywords:** parotid neoplasms, sarcopenia, muscle, prognosis

## Abstract

**Background:**

Parotid gland carcinoma (PGC) is rare and prognostic evidence is limited, making preoperative risk stratification challenging. We evaluated the prognostic relevance of pretreatment skeletal muscle mass measured on routine cervical magnetic resonance imaging.

**Methods:**

We retrospectively analyzed 58 patients with histopathologically confirmed PGC treated between 1991 and 2024. Cervical skeletal muscle mass was quantified as the C3 skeletal muscle index (C3SMI) using axial *T*_2_-weighted magnetic resonance imaging (MRI) at the C3 level. Overall survival (OS) and recurrence-free survival (RFS) were assessed using Kaplan–Meier analysis and Cox proportional hazards models.

**Results:**

Low C3SMI was significantly associated with worse OS and RFS on Kaplan–Meier analysis. In multivariable Cox models restricted to pretreatment variables, low C3SMI remained an independent predictor of shorter OS and RFS. In addition, low C3SMI was significantly associated with adverse pathological features, including high-grade histology, perineural invasion, extracapsular extension, and lymphovascular invasion.

**Conclusions:**

MRI-derived C3SMI is an independent prognostic biomarker for survival and recurrence in PGC and correlates with pathological aggressiveness. Because C3SMI can be obtained from standard preoperative cervical MRI without additional imaging or radiation exposure, it may provide a practical tool for preoperative risk stratification and treatment planning in patients with PGC.

## Teaser

MRI-derived C3 skeletal muscle index independently predicts survival and recurrence in parotid gland carcinoma, providing a practical preoperative biomarker for risk stratification.

## Introduction

Malignant tumors of the parotid gland are rare, accounting for ~0.3% of all human cancers [1] and 1%–3% of head and neck malignancies [2]. Their rarity, combined with marked histopathological heterogeneity, poses considerable challenges for prognosticating survival outcomes and planning treatment.

For parotid gland carcinoma (PGC), reported 10-year disease-specific survival rates in major centers range from ~47% to 69%, depending on patient population and tumor characteristics [3]. Given this variability, accurate estimation of prognosis prior to surgery is particularly important. Several studies have investigated pretreatment prognostic factors and developed risk-prediction models based solely on variables available before surgery [4–6]. Reliable preoperative prognostication of survival outcomes can assist clinicians in tailoring the treatment intensity, optimizing surgical strategies, and planning appropriate follow-up. Moreover, providing individualized prognostic information facilitates shared decision-making and supports risk stratification in clinical practice.

In recent years, pretreatment skeletal muscle mass (SMM) has gained increasing recognition as a novel prognostic indicator across various malignancies, including head and neck cancers (HNCs) [7–9]. Low pretreatment SMM has been associated with impaired tolerance to oncologic therapies, heightened systemic inflammation, and ultimately inferior survival outcomes [10]. However, despite increasing evidence in other tumor entities, the prognostic relevance of SMM in PGC remains largely unexplored.

Given that magnetic resonance imaging (MRI) of the cervical region is commonly performed in patients with parotid gland tumors, it offers a unique opportunity to evaluate SMM preoperatively at the level of the third cervical vertebra (C3). The C3 skeletal muscle index (C3SMI) has been validated as a surrogate for whole-body muscle mass and may thus provide clinically meaningful prognostic information without requiring additional imaging or radiation exposure [11, 12]. Because MRI is often included in the preoperative evaluation, C3SMI measurement could serve as a practical and straightforward tool for pretreatment risk stratification in patients with PGC.

The present study, therefore, aimed to evaluate various preoperative factors associated with prognosis and, in particular, to investigate the prognostic impact of pretreatment C3SMI measured on MRI on overall survival (OS) and recurrence-free survival (RFS) in patients with PGC.

## Material and methods

### Study population

This retrospective study included patients with histopathologically confirmed PGC who received treatment between June 1991 and July 2024. A total of 157 patients were initially identified. Patients with distant metastasis (M1) at diagnosis and those without available preoperative MRI images were excluded. In addition, patients with sebaceous carcinoma were excluded because this histological subtype most commonly represents parotid lymph node metastasis from cutaneous malignancies rather than a primary PGC, leaving 58 patients for the final analysis.

### Data collection

Clinical and pathological data were retrospectively collected from electronic and paper-based medical records, depending on the treatment period. The variables included patient demographics (age, sex) and clinical features (facial nerve paralysis, pain or tenderness, adhesion or immobility, and MRI-defined tumor margin), as well as clinical tumor classification (cT, cN, and cTNM stage). In addition, laboratory data were reviewed to calculate the neutrophil-to-lymphocyte ratio (NLR) as a systemic inflammatory marker. Postoperative pathological findings, including histological type, histological grade, surgical margin status, perineural invasion (PNI), extracapsular extension (ECE), and lymphovascular invasion (LVI), were retrieved.

Pain/tenderness was defined as the presence of spontaneous pain or tenderness on palpation in the parotid region at initial presentation, as documented in the clinical records.

Adhesion/immobility was defined as reduced mobility of the tumor relative to surrounding tissues on physical examination, suggesting adhesion or fixation, as recorded in the clinical notes.

MRI-defined margin was classified based on pretreatment MRI findings as either well-defined or ill-defined, according to whether the tumor margin could be clearly distinguished from adjacent tissues on *T*_2_-weighted and contrast-enhanced images.

### Treatment

The majority of patients underwent surgical resection as the initial treatment (partial, total, or extended total parotidectomy, with or without concomitant neck dissection), whereas a subset of patients underwent only biopsy without definitive surgery. When the tumor directly invaded the facial nerve, it was resected; otherwise, anatomical preservation was prioritized. Neck dissection was simultaneously performed in cases with clinically or radiologically evident nodal metastases.

Postoperative radiotherapy or chemoradiotherapy was indicated for patients exhibiting adverse pathological factors, such as high histological grade, PNI or LVI, nodal involvement, and close or positive resection margins. Radiation therapy was typically delivered at 2.0 Gy per fraction, 5 days per week, to a total dose of 50–60 Gy, although individual adjustments were made based on patient performance status and comorbid conditions. Patients with distant metastases at initial diagnosis or those with unresectable disease underwent only diagnostic biopsy, followed by systemic chemotherapy as the primary treatment.

### Histological type and grade

The histological types and grades of PGC in this study were reviewed and re-evaluated according to the World Health Organization (WHO) Classification of Head and Neck Tumors, 5th Edition [13]. For tumor entities not graded by the WHO criteria, the histological grade was defined as follows. Salivary duct carcinoma, squamous cell carcinoma, and carcinosarcoma were regarded as high-grade, whereas basal cell adenocarcinoma, sebaceous adenocarcinoma, and lymphoepithelial carcinoma were classified as non-high-grade tumors, and acinic cell carcinoma and secretory carcinoma were categorized as low-grade. Mucoepidermoid carcinoma and adenocarcinoma not otherwise specified were graded according to the WHO criteria and further dichotomized into high-grade versus non-high-grade groups. Adenoid cystic carcinoma was classified into tubular, cribriform, and solid types based on the predominant histological growth pattern; tumors with a solid component were considered high-grade tumors [14]. For carcinoma ex pleomorphic adenoma, grade was determined according to the histological type and grade of the malignant component. Cases demonstrating dedifferentiation or high-grade transformation were classified as high-grade.

### MRI acquisition

MRI was performed using 1.5- or 3.0-T scanners with ≥16-channel head-and-neck coils. Axial *T*_2_-weighted images for C3SMI measurement were obtained with typical parameters: repetition time (TR) 3000–6000 ms, echo time (TE) 70–110 ms, slice thickness 4–5 mm, interslice gap 0.4–1.0 mm, field of view 200–240 mm, and matrix 320 × 224 to 384 × 288. Additional sequences (*T*_1_-weighted, diffusion-weighted, and postcontrast imaging) were acquired according to institutional protocols.

### Assessment of skeletal muscle mass

**Figure 1:**
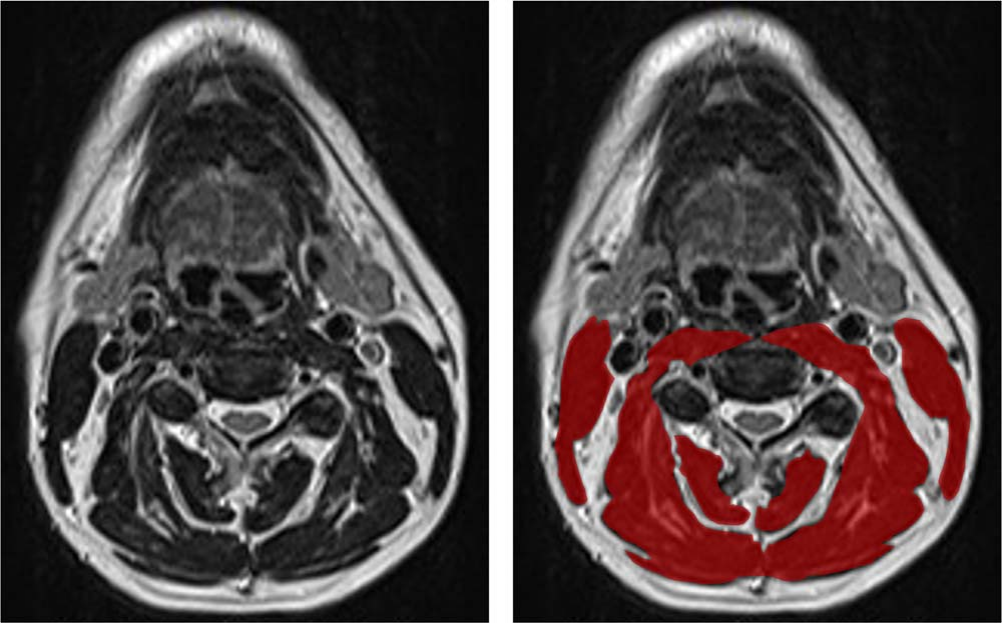
Paravertebral and sternocleidomastoid muscle area measurement at the level of the third cervical vertebra (C3) on axial *T*_2_-weighted imaging (T2WI). The left panel shows unsegmented muscle tissue, whereas the right panel illustrates the segmented paravertebral and sternocleidomastoid muscles highlighted in red. Abbreviation: MRI, magnetic resonance imaging.

Pretreatment cervical MRI was used to estimate SMM. Following the method of Zwart and Swartz, the skeletal muscle area was measured on ImageJ (NIH, Bethesda, MD, USA) using the first axial *T*_2_-weighted image that clearly displayed the C3 vertebra [12, 15]. The bilateral sternocleidomastoid and paraspinal muscles were manually outlined, and their combined cross-sectional area (CSA) was obtained (Fig. 1). The C3SMI was calculated by normalizing the CSA to patient height:


$$ \mathrm{C}3\mathrm{SMI}\;\left({\mathrm{cm}}^{{}^2}/{\mathrm{m}}^{{}^2}\right)=\mathrm{C}3\ \mathrm{CSA}\ \left({\mathrm{cm}}^{{}^2}\right)\div{\mathrm{Height}}^{{}^2}\ \left({\mathrm{m}}^{{}^2}\right) $$

Patients were dichotomized into low- and high-LSMI groups using sex-specific C3SMI cutoff values. Low LSMI was defined as a C3SMI < 12.83 cm^2^/m^2^ for men and <11.15 cm^2^/m^2^ for women, in accordance with a previously published MRI-based study that determined optimal thresholds using receiver operating characteristic curve analysis with the Youden index [11].

### Statistical analysis

All the statistical analyses were performed using SPSS Statistics 30.0 (IBM Corp.). Continuous variables were summarized as medians with interquartile ranges, and categorical variables as frequencies and percentages. OS and RFS were defined from initial treatment to death/last follow-up and to first recurrence, respectively. Survival curves were generated using the Kaplan–Meier method and compared with the log-rank test. Prognostic factors for OS and RFS were evaluated using Cox proportional hazards regression analyses. Given the limited number of events, multivariable models were constructed using clinically relevant preoperative variables selected a priori, rather than based on univariate screening. The number of covariates included in each multivariable model was restricted according to the number of events, and overlapping variables were not simultaneously included to avoid model instability. Separate multivariable models were constructed for OS and RFS. Pretreatment clinical factors were compared according to pathological outcomes using the chi-square or Fisher’s exact test for categorical variables and the Mann–Whitney *U* test for continuous variables. A two-sided *P* < .05 was considered statistically significant.

This retrospective study was approved by the institutional review board (approval number: 20190093) and was conducted in accordance with the ethical standards of the institutional research committee and with the Helsinki Declaration of 1975, as revised in 1983. Because of the retrospective nature of the study, the requirement for written informed consent was waived, and an opt-out approach was adopted.

## Results

### Patient characteristics

A total of 58 patients with PGC were included in the present analysis. The baseline clinical characteristics are presented in Table 1. The cohort comprised 33 males and 25 females, yielding a male-to-female ratio of 1.3. The median pretreatment age was 57 years (interquartile range, 45–68 years).

**Table 1:** Patient and disease characteristics.

		Total		Low C3SMI		High C3SMI		*P*-value
		*n* = 58	%	*n* = 21	%	*n* = 37	%	
Age	Median (IQR)	57 (45–68)		56 (42–67)		56 (43–65)	–	.785
Sex	Male/Female	33/25	56.9/43.1	6/15	28.6/71.4	19/18	51.4/48.6	.092
T classification	T1/T2/T3/T4	22/15/6/15	37.9/25.9/10.3/25.9	4/4/2/11	19.0/19.0/9.5/52.4	18/11/4/4	48.6/29.7/10.8/10.8	**.005**
N classification	N0/N1/N2/N3	47/2/7/2	81.0/3.4/12.1/3.4	11/2/6/2	52.4/9.5/28.6/9.5	36/0/1/0	97.3/0.0/0.0/2.7	**<.001**
TNM stage	I/II/III/IV	19/14/10/15	32.8/24.1/17.2/25.9	1/3/6/11	4.8/14.3/28.6/52.4	18/11/4/4	48.6/29.7/10.8/10.8	**<.001**
Facial nerve paralysis	Yes/No	9/49	15.5/84.5	4/17	19.0/81.0	5/32	13.5/86.5	.576
Pain/Tenderness	Yes/No	20/38	34.5/65.5	9/12	42.9/57.1	11/26	29.7/70.3	.312
Adhesion/Immobility	Yes/No	24/34	41.4/58.6	14/7	66.7/33.3	10/27	27.0/73.0	**.003**
NLR	Median (IQR)	1.94 (1.38–2.90)		1.73 (1.11–2.33)		2.06 (1.57–3.05)	–	.172
MRI-defined margin*	Clear/Indistinct	28/30	49.1/50.9	4/16	19.0/81.0	24/13	64.9/35.1	**.001**
C3SMI (cm^2^/m^2^)	Median (IQR)	13.53 (10.32–16.93)		9.63 (8.09–10.94)		16.22 (13.53–18.40)		**<.001**
Surgical margin^a^	Positive/Negative	18/38	32.1/67.9	11/9	57.1/42.9	7/29	18.9/78.4	**.006**
Perineural invasion^a^	Yes/No	21/32	36.2/55.2	11/7	52.4/33.3	10/25	27.0/67.6	**.022**
Extracapsular extension*	Yes/No	8/37	13.8/63.8	7/12	33.3/57.1	1/25	2.7/67.6	**.004**
Lymphovascular invasion*	Yes/No	19/20	32.8/34.5	7/5	33.3/23.8	0/21	0/56.8	**<.001**

Abbreviations: C3SMI, cervical level 3 skeletal muscle index; IQR, interquartile range; MRI, magnetic resonance imaging; NLR, neutrophil-to-lymphocyte ratio.

Bold values indicate statistically significant differences between groups.

^a^Data were missing for five variables due to incomplete medical records; percentages and means were calculated based on available cases. Bold values indicate statistically significant differences between groups. Data were missing for five variables due to incomplete medical records; percentages and means were calculated based on available cases.

Based on pathological tumor–node–metastasis (TNM) classification, 21 patients presented with locally advanced primary tumors (T3–T4), 11 exhibited cervical lymph node involvement, and 25 were classified as having stage III–IV disease overall. The clinical symptoms included facial nerve paralysis in 9 patients, pain or tenderness in 20, and adhesion or immobility in 24. According to the pathological findings, high-grade tumors were identified in 30 patients (51.7%). Positive surgical margins were present in 18 patients (31.0%), PNI was observed in 21 cases (36.2%), ECE was detected in 8 cases (13.8%), and LVI was evident in 19 cases (32.8%). However, data for these pathological variables were missing in a subset of patients, and the analyses involving these factors were performed based on the available cases.

### Histological type and grade

Histopathological characteristics of patients with PGC are summarized in Table 2. Among the histological types, carcinoma ex pleomorphic adenoma was the most frequent (*n* = 12), followed by mucoepidermoid carcinoma (*n* = 11), salivary duct carcinoma (*n* = 9), acinic cell carcinoma (*n* = 7), and basal cell adenocarcinoma (*n* = 7). None of the PGC cases demonstrated dedifferentiation or high-grade transformation. Overall, 28 tumors were classified as low- or intermediate-grade, whereas 30 were classified as high-grade.

**Table 2:** Histological type and grade of parotid grand tumor.

	Case (*n* = 58)			%
Histological type	Low or intermediate grade	High grade	Overall	
Overall	28	30	58	100
Carcinoma ex pleomorphic adenoma	2	9	11	19.0
Mucoepidermoid carcinoma	11		11	19.0
Salivary duct carcinoma		9	9	15.5
Acinic cell carcinoma	7		7	12.1
Basal cell adenocarcinoma	7		7	12.1
Squamous cell carcinoma		6	6	10.3
Adenocarcinoma, not otherwise specified	1	3	4	6.9
Secretory carcinoma		2	2	3.4
Adenoid cystic carcinoma		1	1	1.7

### OS and RFS in the entire cohort

During a median follow-up period of 64 months (range, 3.6–192 months), the 5-year OS rate was 83.2%, and the 5-year RFS rate was 71.6%. As shown in Supplementary Fig. 1, neither the median OS nor the median RFS was reached during the follow-up period, indicating generally favorable long-term outcomes in this cohort.

### Comparison of OS and RFS between low- or intermediate-grade and high-grade tumors

**Figure 2:**
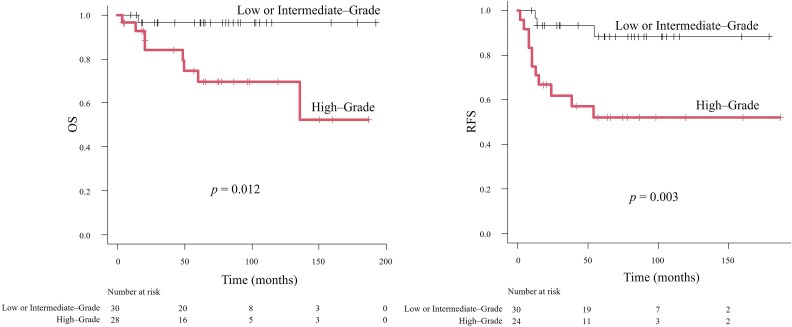
Kaplan–Meier curves for OS and RFS according to histological grade (low/intermediate grade vs high grade). Abbreviations: OS, overall survival; RFS, recurrence-free survival.

Kaplan–Meier analysis was conducted to compare survival outcomes between patients with intermediate-grade and high-grade tumors. Patients with high-grade tumors had significantly shorter OS (*P* = .012) and RFS (*P* = .003) than those with intermediate-grade tumors (Fig. 2).

### Pretreatment prognostic factors for OS and RFS

To identify potential prognostic factors for OS, univariate analysis was conducted focusing on pretreatment clinical variables to predict survival outcomes prior to surgery (Table 3). The analysis demonstrated that baseline TNM stage [*P* = .019, hazard ratio (HR): 6.84, 95% confidence interval (CI) 1.37–34.19], and C3SMI (*P* = .005, HR: 9.64, 95% CI 1.97–47.31) were significantly associated with OS. In the multivariate analysis, only C3SMI (*P* = .037, HR: 6.23, 95% CI 1.12–34.58) remained an independent prognostic factor for OS.

**Table 3:** Prognostic factors for OS in the univariate and multivariate analysis, restricted to variables available prior to treatment.

Variables		Univariate		Multivariate	
		HR (95% CI)	*P*-value	HR (95% CI)	*P*-value
Age	≧65/<65	2.49 (0.62–9.97)	.197		
Sex	Male/Female	1.46 (0.37–5.86)	.590		
TNM stage	III–IV/I–II	6.84 (1.37–34.19)	**.019**	3.68 (0.59–22.91)	.162
Facial nerve paralysis	Yes/No	1.72 (0.36–8.27)	.500		
Pain/Tenderness	Yes/No	2.46 (0.66–9.20)	.181		
Adhesion/Immobility	Yes/No	2.03 (0.54–7.59)	.294		
NLR	High/Normal	0.22 (0.05–1.08)	.061		
MRI-defined margin	Indistinct/Clear	68.27 (0.32–14602.304)	.123		
C3SMI (cm^2^/m^2^)	Low/Normal	9.64 (1.97–47.31)	**.005**	6.23 (1.12–34.58)	**.037**

Abbreviations: CI, confidence interval; C3SMI, cervical level 3 skeletal muscle index; HR, hazard ratio; OS, overall survival; MRI, magnetic resonance imaging; NLR, neutrophil-to-lymphocyte ratio.

Bold values indicate statistically significant differences between groups.

We then performed a similar analysis for RFS, focusing on pretreatment clinical variables, to identify prognostic factors predictive of recurrence prior to surgery. (Table 4). In the univariate analysis, baseline age (*P* = .044, HR: 2.94, 95% CI 1.03–8.41), TNM stage (*P* = .018, HR: 3.75, 95% CI 1.25–11.22), pain or tenderness (*P* = .024, HR: 3.37, 95% CI 1.17–9.73), adhesion or immobility (*P* = .020, HR: 3.67, 95% CI 1.23–10.97), NLR (*P* = .009, HR: 0.18, 95% CI 0.05–0.66), MRI-defined margin (*P* = 0.010, HR: 7.20, 95% CI 1.61–32.24), and C3SMI (*P* < 0.001, HR: 11.99, 95% CI 3.30–43.55) were significantly associated with RFS. In the multivariate analysis, only C3SMI (*P* = .012, HR: 6.56, 95% CI 1.52–28.30) remained an independent prognostic factor for RFS.

**Table 4:** Prognostic factors for RFS in the univariate and multivariate analysis, restricted to variables available prior to treatment.

Variables		Univariate		Multivariate	
		HR (95% CI)	*P*-value	HR (95% CI)	*P*-value
Age	≧65/<65	2.94 (1.03–8.41)	**.044**		
Sex	Male/Female	1.35 (0.45–4.02)	.594		
TNM stage	III-IV/I-II	3.75 (1.25–11.22)	**.018**	1.77 (0.51–6.12)	.364
Facial nerve paralysis	Yes/No	2.05 (0.57–7.34)	.273		
Pain/Tenderness	Yes/No	3.37 (1.17–9.73)	**.024**		
Adhesion/Immobility	Yes/No	3.67 (1.23–10.97)	**.020**		
NLR	High/Normal	0.18 (0.05–0.66)	**.009**	0.27 (0.07–1.06)	.060
MRI-defined margin	Indistinct/Clear	7.20 (1.61–32.24)	**.010**		
C3SMI (cm^2^/m^2^)	Low/Normal	11.99 (3.30–43.55)	**<.001**	6.56 (1.52–28.30)	**.012**

Abbreviations: CI, confidence interval; C3SMI, cervical level 3 skeletal muscle index; HR, hazard ratio; MRI, magnetic resonance imaging; NLR, neutrophil-to-lymphocyte ratio; RFS, recurrence-free survival.

Bold values indicate statistically significant differences between groups.

### Comparison of OS and RFS between low- and normal-C3SMI groups

**Figure 3:**
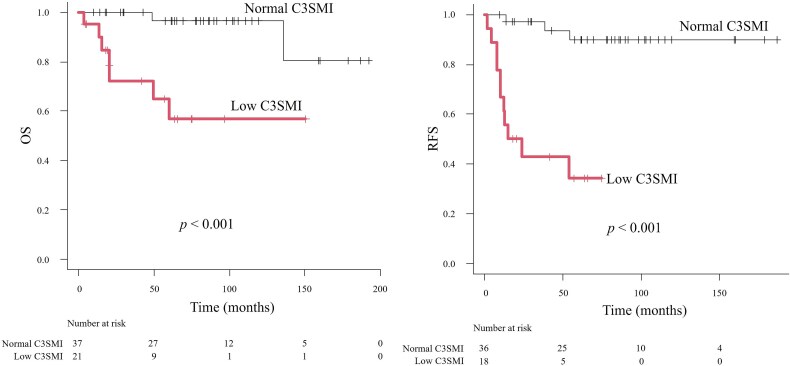
Kaplan–Meier curves for OS and RFS according to C3SMI. Abbreviations: C3SMI, cervical level 3 skeletal muscle index; OS, overall survival; RFS, recurrence-free survival.

Kaplan–Meier analysis was conducted to compare survival outcomes between patients with low and normal C3SMI. Patients with low C3SMI exhibited significantly shorter OS (*P* < .001) and RFS (*P* < .001) compared with those with normal C3SMI (Fig. 3).

### Comparison of pretreatment characteristics according to postoperative pathological outcomes

We also compared pretreatment clinical factors between groups stratified by postoperative pathological findings. As shown in Table 5, patients with high-grade tumors were significantly more likely to be male and to present with higher T classification (T3–4), nodal involvement (N1–3), and advanced TNM stage (III–IV). In addition, adhesion or immobility, ill-defined MRI margins, and low C3SMI were all more frequently observed in the high-grade group than in the low/intermediate-grade group.

**Table 5:** Comparison of pretreatment characteristics between low-/intermediate-grade and high-grade tumors.

Variables		Low or intermediate grade	High grade	*P*-value
Age	≧65/<65	9/21	9/19	.860
Sex	Male/Female	13/17	20/8	**.031**
T classification	1–2/3–4	24/6	13/15	**.008**
N classification	0/1–3	29/1	15/13	**<.001**
TNM stage	I-II/III-IV	24/6	9/19	**<.001**
Facial nerve paralysis	Yes/No	3/27	6/22	.230
Pain/Tenderness	Yes/No	7/23	13/15	.064
Adhesion/Immobility	Yes/No	6/24	18/10	**<.001**
NLR	High/Low	18/12	14/14	.444
MRI-defined margin	Indistinct/Clear	10/20	20/8	**.004**
C3SMI (cm^2^/m^2^)	Low/Normal	1/29	20/8	**<.001**

Abbreviations: C3SMI, cervical level 3 skeletal muscle index; MRI, magnetic resonance imaging; NLR, neutrophil-to-lymphocyte ratio.

Bold values indicate statistically significant differences between groups.

Comparisons for other pathological features, including PNI, ECE, and LVI, are presented in Supplementary Tables 1–3. Several pretreatment factors showed statistically significant associations with these pathological findings, and the proportion of patients with low C3SMI was higher in the PNI-positive, ECE-positive, and LVI-positive groups.

## Discussion

Various pretreatment factors have been identified as potential prognostic indicators in PGC. The prognostic significance of T, N, and M classifications has been established [16–19]. In addition, several clinical and hematological factors, including facial nerve paralysis [20], pain or tenderness [21], adhesion or immobility [22], pretreatment lymphocyte-to-monocyte ratio [23], and ill-defined tumor margins on MRI [5], are associated with poor prognosis. These factors, readily assessable through routine examination and imaging, may thus serve as practical and straightforward prognostic markers.

In addition to these clinical and imaging-based factors, low SMM has recently been recognized as an important negative prognostic factor across various cancer types, including HNCs [8, 9]. In a cohort of 74 patients with salivary gland carcinoma (SGC), Vincenzo Abbate et al. [24] demonstrated that low SMM was associated with significantly worse OS and disease-specific survival; however, the absence of multivariate analyses, such as Cox proportional hazards modeling, limited the robustness of their conclusions. To date, this remains the only study specifically addressing the prognostic role of SMM in SGC. Given the scarcity of data regarding SMM in the SGC field, our study provides novel evidence by focusing specifically on PGC, in which low SMM emerged as an independent adverse prognostic factor for both OS and RFS. The underlying mechanisms linking low SMM to poor prognosis have not been fully elucidated; however, skeletal muscle is increasingly recognized not only as a structural tissue but also as an active secretory organ that secretes immunomodulatory cytokines, known as myokines [25]. These myokines includiong irisin [26, 27], decorin [28, 29], interleukin-15 [25, 30, 31], secreted protein acidic and rich in cysteine (SPARK) [32], oncostatin M [33], and brain-derived neurotrophic factor (BDNF) [34, 35]—have been shown to enhance antitumor immune responses by promoting T-cell and natural killer cell activity, suppressing oncogenic signaling, and inhibiting tumor progression. Accordingly, diminished SMM may impair myokine-mediated immunosurveillance, thereby contributing to worse oncologic outcomes. Moreover, chronic systemic inflammation—commonly present in cancer—may contribute to both muscle depletion and tumor progression, suggesting that low SMM serves as a surrogate marker of biologically aggressive disease.

In addition, the proportion of patients with low C3SMI was significantly higher among those with high-grade tumors, as well as in the PNI-positive, ECE-positive, and LVI-positive groups, indicating that reduced SMM tends to be associated with more aggressive pathological features. This finding supports the notion that low SMM reflects aggressive tumor biology rather than only host frailty. Reduced SMM may indicate systemic inflammation and catabolism, and may also mirror tumor–host interactions that promote invasive growth. Indeed, prior studies have shown that low SMM correlates with elevated inflammatory markers and tumor-associated metabolic stress [10, 36]. Collectively, these results suggest that MRI-derived C3SMI is associated with high-grade tumors and positive pathological features such as PNI, ECE, and LVI, which may partly account for its link to poor prognosis in PGC.

In our study, none of the evaluated pretreatment factors, including T and N classifications, pain/tenderness, facial nerve paralysis, adhesion or immobility, and MRI-defined margin, reached statistical significance for OS or RFS in the multivariate analysis, in contrast to previous reports. These discrepancies between our findings and previous reports may, at least in part, be explained by differences in study design and patient composition. Our cohort comprised 58 patients encompassing all histological types of PGC, which naturally exhibit marked heterogeneity in biological behavior, patterns of invasion, and metastatic potential. In contrast, many prior studies focused on a single type—such as salivary duct carcinoma, carcinoma ex pleomorphic adenoma, or adenoid cystic carcinoma—wherein clinicopathologic features such as pain, facial nerve paralysis, and MRI-defined margins may more consistently reflect tumor aggressiveness. This histological diversity in our series may have attenuated the prognostic impact of certain pretreatment indicators.

In the management of PGC, the ability to identify patients at high risk of poor outcomes based on pretreatment factors is of substantial clinical significance. Unlike postoperative pathological findings, which become available only after surgery, pretreatment variables allow clinicians to stratify patients before initiating therapy. This early risk stratification can support timely optimization of surgical strategies, proactive consideration of adjuvant treatment, and individualized surveillance planning. Furthermore, early recognition of patients with low SMM allows implementation of perioperative nutritional and rehabilitative interventions to mitigate complications, while also enabling more informed discussions with patients and their families regarding treatment risks, prognosis, and shared decision-making. Thus, prognostic assessment based on pretreatment factors, such as MRI-derived SMM, may contribute to more personalized and effective management of PGC.

Several limitations of this study should be acknowledged. It was conducted using data extracted from the institution’s electronic medical records as well as paper-based clinical records. Consequently, the cohort may be influenced by selection bias. In addition, the investigation was limited to cases from a single center, which could introduce epidemiological bias. Furthermore, because the present study included various histological types of PGC analyzed collectively, the inherent heterogeneity in tumor biology and treatment response may have confounded the prognostic impact of certain factors. In particular, the proportion of adenoid cystic carcinoma cases was relatively small, which may have limited the generalizability of the results to this histological type. To validate these preliminary findings, prospective multicenter studies with larger sample sizes and longer follow-up periods will be necessary. Moreover, although MRI-based estimation of SMM is increasingly utilized, the methodology has not yet been fully standardized, which should also be acknowledged as a limitation of this study.

## Conclusion

Low SMM assessed by C3SMI on pretreatment MRI was an independent predictor of both OS and RFS in patients with PGC. Low C3SMI also correlated with pathological markers of tumor aggressiveness, indicating that reduced muscle mass may reflect adverse tumor biology. C3SMI, easily derived from routine MRI, represents a simple and clinically useful tool for preoperative risk stratification and treatment planning.

## Supplementary Material

Supplementary_materials_hyag033
