# Supplementary material for: Prognostic impact of MRI-based cervical skeletal muscle mass on survival in parotid gland carcinoma
Source: Jpn J Clin Oncol. 2026 Mar 2;56(6):713–21. doi: 10.1093/jjco/hyag033 (PMC13237869; doi:10.1093/jjco/hyag033)
Supplement: Supplementary_materials_hyag033 [file supplementary_materials_hyag033.zip › (Supplementary_Tables)_for_JJCO_0128_hyag033.docx]

Supplementary table 1. Comparison of pre-treatment characteristics between patients with and without PNI among patients with available PNI data (n = 53).

*Abbreviations: PNI (perineural invasion); NLR (neutrophil-to-lymphocyte ratio); MRI (magnetic resonance imaging); C3SMI (cervical level 3 skeletal muscle index).*

| **variables** |  | **PNI (-)** | **PNI (+)** | ***p*-value** |
| --- | --- | --- | --- | --- |
| Age | ≧65 / <65 | 6/26 | 10/11 | **0.025** |
| Sex | Male / Female | 17/15 | 14/7 | 0.328 |
| T classification | 1-2 / 3-4 | 28/4 | 7/14 | **< 0.001** |
| N classification | 0 / 1-3 | 26/6 | 14/7 | 0.227 |
| TNM stage | I-II / III-IV | 27/5 | 5/16 | **< 0.001** |
| Facial nerve paralysis | Yes / No | 0/32 | 7/14 | **< 0.001** |
| Pain/tenderness | Yes / No | 7/25 | 10/11 | 0.050 |
| Adhesion/immobility | Yes / No | 7/25 | 13/8 | **0.003** |
| NLR | High /Low | 18/14 | 11/10 | 0.782 |
| MRI-defined margin | Indistinct / Clear | 11/21 | 16/5 | **0.003** |
| C3SMI (cm^2^/m^2^) | Low / Normal | 7/25 | 11/10 | **0.022** |

Supplementary Table 2. Comparison of pre-treatment characteristics between patients with and without ECE among patients with available ECE data (n = 45).

*Abbreviations: ECE (extracapsular extension); NLR (neutrophil-to-lymphocyte ratio); MRI (magnetic resonance imaging); C3SMI (cervical level 3 skeletal muscle index).*

| **variables** |  | **ECE (-)** | **ECE (+)** | ***p*-value** |
| --- | --- | --- | --- | --- |
| Age | ≧65 / <65 | 13/24 | 2/6 | 0.581 |
| Sex | Male / Female | 19/18 | 8/0 | **0.011** |
| T classification | 1-2 / 3-4 | 29/8 | 0/8 | **< 0.001** |
| N classification | 0 / 1-3 | 30/7 | 3/5 | **0.011** |
| TNM stage | I-II / III-IV | 25/12 | 0/8 | **< 0.001** |
| Facial nerve paralysis | Yes / No | 4/33 | 3/5 | 0.059 |
| Pain/tenderness | Yes / No | 11/26 | 3/5 | 0.667 |
| Adhesion/immobility | Yes / No | 14/23 | 6/2 | 0.055 |
| NLR | High / Low | 20/17 | 4/4 | 0.835 |
| MRI-defined margin | Indistinct / Clear | 16/21 | 7/1 | **0.023** |
| C3SMI (cm^2^/m^2^) | Low / Normal | 12/27 | 7/1 | **0.004** |

Supplementary Table 3. Comparison of pre-treatment characteristics between patients with and without LVI among patients with available LVI data (n = 39).

*Abbreviations: LVI (lymphovascular invasion); NLR (neutrophil-to-lymphocyte ratio); MRI (magnetic resonance imaging); C3SMI (cervical level 3 skeletal muscle index).*

| **variables** |  | **LVI (-)** | **LVI (+)** | **p-value** |
| --- | --- | --- | --- | --- |
| Age | ≧65 / <65 | 5/15 | 9/10 | 0.146 |
| Sex | Male / Female | 10/10 | 11/8 | 0.621 |
| T classification | 1-2 / 3-4 | 18/2 | 8/11 | **0.002** |
| N classification | 0 / 1-3 | 18/2 | 11/8 | **0.022** |
| TNM stage | I-II / III-IV | 17/3 | 7/12 | **0.002** |
| Facial nerve paralysis | Yes / No | 1/19 | 3/16 | 0.267 |
| Pain/tenderness | Yes / No | 5/15 | 9/10 | 0.146 |
| Adhesion/immobility | Yes / No | 5/15 | 11/8 | **0.037** |
| NLR | High /low | 15/5 | 6/13 | **0.007** |
| MRI-defined margin | Indistinct / Clear | 5/15 | 12/7 | **0.016** |
| C3SMI (cm^2^/m^2^) | Low / Normal | 1/19 | 13/6 | **< 0.001** |
