# Supplementary figures and images for: Prognostic impact of MRI-based cervical skeletal muscle mass on survival in parotid gland carcinoma
Source: Jpn J Clin Oncol. 2026 Mar 2;56(6):713–21. doi: 10.1093/jjco/hyag033 (PMC13237869; doi:10.1093/jjco/hyag033)

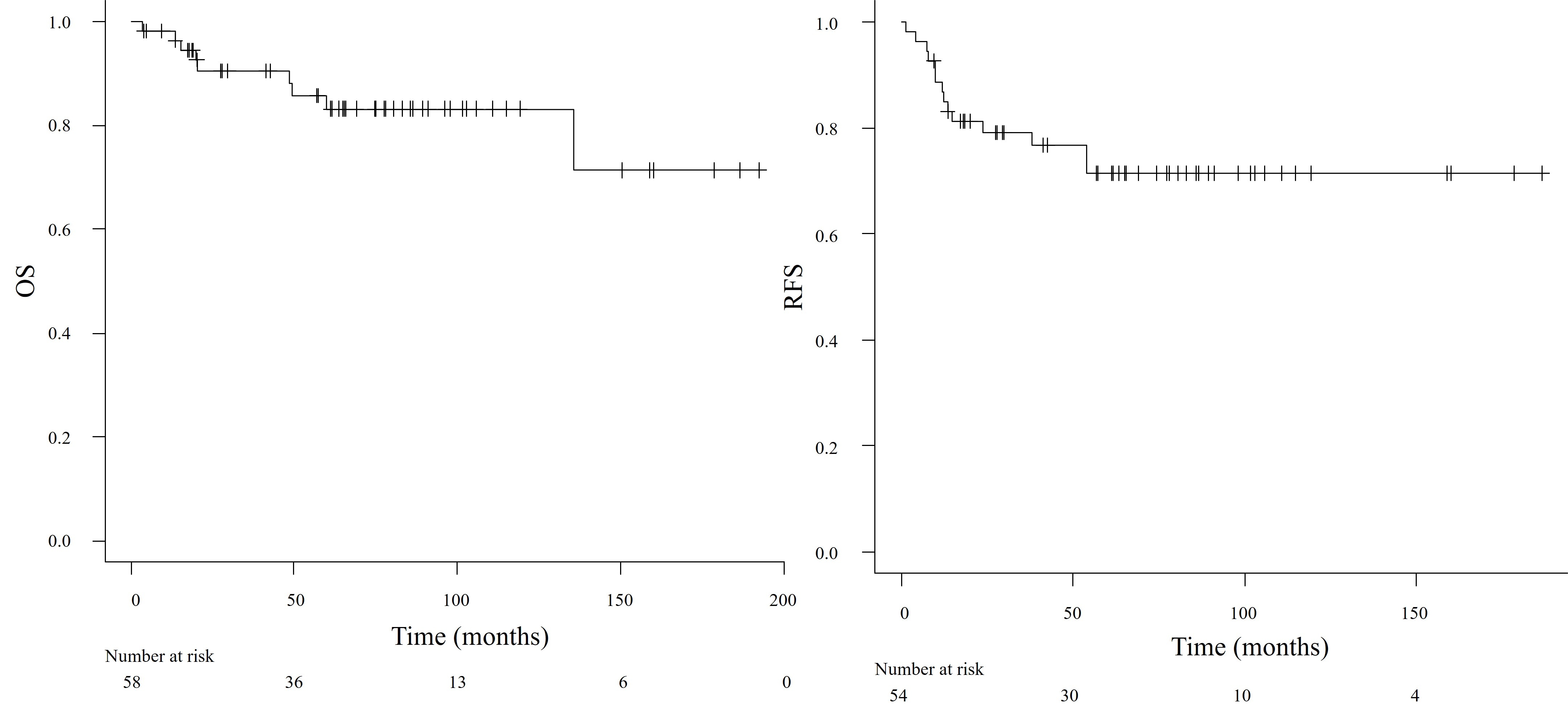

Supplement: Supplementary_materials_hyag033 [file supplementary_materials_hyag033.zip › Supplementary Figure 1.jpg]
